# Correction: Enhanced visible light photocatalytic activity of a floating photocatalyst based on B–N-codoped TiO_2_ grafted on expanded perlite

**DOI:** 10.1039/d2ra90092k

**Published:** 2022-09-23

**Authors:** Xin Wang, Wei Wang, Xuejiang Wang, Jing Zhang, Zaoli Gu, Lijie Zhou, Jianfu Zhao

**Affiliations:** College of Environmental Science and Engineering, State Key Laboratory of Pollution Control and Resource Reuse, Tongji University Shanghai 200092 China wangxj@tongji.edu.cn +86 021 65984268

## Abstract

Correction for ‘Enhanced visible light photocatalytic activity of a floating photocatalyst based on B–N-codoped TiO_2_ grafted on expanded perlite’ by Xin Wang *et al.*, *RSC Adv.*, 2015, **5**, 41385–41392, https://doi.org/10.1039/c5ra06056g.

The Royal Society of Chemistry has been made aware of a closely related paper, published by the authors at nearly the same time in *Applied Surface Science*.^1^ Although different data and results are reported in each article, the *Applied Surface Science* article should have been cited in this *RSC Advances* article.

The authors have been contacted but have not responded to our communication.

The Royal Society of Chemistry apologises for these errors and any consequent inconvenience to authors and readers.

## Supplementary Material
